# Differences in International Recommendations for Piperacillin Dosing: A Japanese Perspective

**DOI:** 10.31662/jmaj.2025-0414

**Published:** 2025-11-28

**Authors:** Osamu Uemura

**Affiliations:** 1 Department of Pediatrics, Ichinomiya Medical Treatment & Habilitation Center, Ichinomiya-city Aichi, Japan

**Keywords:** Sanford Guide, Japanese package insert, piperacillin, pharmacokinetic, pharmacodynamic, intensive care unit, *Pseudomonas aeruginosa*, general wards

## Abstract

Differences in antibiotic dosing recommendations highlight the tension between maximizing efficacy and ensuring safety. Piperacillin is a striking example, with the Sanford Guide recommending regimens of 13.5-27 g/day based on pharmacokinetic and pharmacodynamic principles aimed at patients who are critically ill, whereas the Japanese package insert advises 2-4 g/day, with a maximum of 16 g/day, reflecting a cautious approach focused on safety in general wards. These divergent strategies reflect distinct philosophies: international guidelines prioritize achieving pharmacokinetic/pharmacodynamic targets to optimize outcomes in patients in intensive care units (ICUs), whereas Japanese labeling emphasizes minimizing adverse events and ecological disruption in broader populations. The clinical consequences of indiscriminate application are significant. High-dose regimens used outside ICU contexts may increase risks of allergy, gastrointestinal disturbance, bone marrow suppression, renal injury, and selection of resistant organisms. Conversely, reliance solely on Japanese recommendations in ICU settings may cause suboptimal exposure and therapeutic failure in severe infections caused by organisms such as *Pseudomonas aeruginosa*. Awareness of these differences is essential for antimicrobial stewardship. Clinicians should tailor dosing to the clinical context, reserving aggressive regimens for patients who are critically ill or at high risk while maintaining safety-oriented dosing in routine ward practice. By recognizing the underlying philosophies and their intended applications, physicians can avoid inappropriate extrapolation, optimize patient outcomes, and reduce the ecological burden of antibiotic use.

## Introduction

Antibiotic dosing is not uniform across health care systems. International guidance documents, national labeling, and institutional protocols often diverge. Although such differences are understandable, they carry implications when recommendations are applied beyond their intended settings. Piperacillin represents a clear example. The Sanford Guide (Sanford Guide 2025) recommends considerably higher doses of piperacillin/tazobactam than the Japanese package insert for piperacillin sodium. This discrepancy is more than a regulatory detail; it reflects contrasting philosophies about efficacy, safety, and target populations. The Japanese context requires particular attention, given clinicians may refer to international resources without fully appreciating the assumptions behind them.

## Piperacillin Dosing in the Sanford Guide

The Sanford Guide 2025 provides regimens such as 3.375 g every 6 hours (total 13.5 g/day) or 4.5 g every 6-8 hours (total 18-27 g/day), and even higher total daily exposures are common when extended or continuous infusions are applied ^(1)^. These recommendations are based on pharmacokinetic/pharmacodynamic (PK/PD) targets, aiming for 100% time above the minimum inhibitory concentration (fT>MIC) ^(2), (3)^. They are intended for intensive care unit (ICU) settings and for serious infections with organisms such as *Pseudomonas aeruginosa*. The emphasis is on maximizing efficacy, particularly in patients who are critically ill with altered drug clearance and distribution. The Sanford approach reflects a philosophy of prioritizing therapeutic success even at the expense of increased drug exposure and potential toxicity.

## Japanese Package Insert Recommendations

In contrast, the Japanese package insert for piperacillin sodium ^(4)^ recommends a daily dose of 2-4 g divided into multiple administrations, with a maximum of 16 g/day in severe cases (Table 1). This dosage reflects expectations of use in general wards, for infections such as community-acquired pneumonia, urinary tract infections, and intestinal infections. It prioritizes safety and tolerability, especially in older patients or those with impaired renal function. Historically, Japan’s regulatory framework has emphasized minimizing adverse reactions, consistent with a philosophy of cautious use in the general patient population. The result is a significant gap between what is recommended internationally and what is considered safe in Japan.

**Table 1.** Comparison of Piperacillin Dosing Recommendations.

Item	Sanford Guide 2025	Japanese package insert
Usual daily dose	3.375 g q6h (13.5 g/day) or 4.5 g q6-8h (18-27 g/day)	2-4 g/day divided into 2-4 doses
Maximum dose	Up to 27 g/day (PIPC/TAZ)	16 g/day
Administration method	Short infusion, extended infusion, or continuous infusion	Short infusion
Clinical context	Patients in ICU, *Pseudomonas*, high-MIC organisms	General ward infections, safety focus

ICU: intensive care unit; MIC: minimum inhibitory concentration; PIPC/TAZ: piperacillin/tazobactam; q6-8h: every 6-8 hours; q6h: every 6 hours.

## Clinical Implications of the Discrepancy

The divergence between these two recommendations creates risks if clinicians apply one framework indiscriminately. In Japanese practice, adopting Sanford-level doses in routine wards could have serious consequences. Higher daily exposures may increase the risk of adverse drug reactions such as drug allergies, gastrointestinal symptoms, bone marrow suppression, and kidney injury. More importantly, excessive dosing in non-ICU contexts can disrupt the microbiological balance, leading to superinfections or selection of resistant organisms, a phenomenon often described as selection of resistant organisms ^(5)^. This is a pressing concern in antimicrobial stewardship, when the balance between sufficient efficacy and minimizing ecological damage is critical. Conversely, adhering strictly to the Japanese package insert in ICU settings may underdose patients who are critically ill with *Pseudomonas aeruginosa* infections, risking treatment failure. Thus, both over-treatment and under-treatment are possible if the clinical context is not considered. The lesson is clear: dosage recommendations cannot be interpreted in isolation; they must be contextualized to the setting, patient population, and therapeutic goals.

## Additional Considerations for Clinical Practice

It is important to recognize that (i) the Sanford Guide dosing recommendations are primarily designed for ICU-level management, particularly when *Pseudomonas aeruginosa* is the causative organism or strongly suspected, or in the setting of severe infections such as sepsis; (ii) piperacillin should not be considered the first-choice treatment for other common infections such as pneumonia, bronchitis, or urinary tract infections, when other antibiotics may be more appropriate; and (iii) if piperacillin is used as a first-choice therapy for these more general infections, dosing should adhere to the Japanese package insert, which is calibrated for safety and general ward contexts. In addition, recent discussions emphasize the role of therapeutic drug monitoring (TDM) and continuous infusion in optimizing piperacillin therapy. TDM allows dose adjustment based on measured serum concentrations, which is especially valuable in patients who are critically ill with altered PKs ^(3)^. Continuous infusion has also been proposed as a means of maximizing fT>MIC, complementing high-dose strategies in ICU contexts ^(2), (5)^. Although these approaches are less common in routine Japanese ward practice, awareness of their potential is important to avoid underdosing in severe infections and to balance efficacy with safety. Recent large clinical studies also addressed continuous infusion strategies ^(6), (7)^. We have therefore highlighted that both TDM and continuous infusion may serve as adjuncts to contextualized dosing strategies.

## Toward Harmonization of Dosing Strategies

Bridging these discrepancies requires transparency and international dialogue. PK/PD principles are universal, but their application depends on context. For patients who are critically ill in ICUs, Sanford-style dosing with extended or continuous infusions may be justified. For patients in routine wards in Japan, however, such regimens are inappropriate and risky. National labeling should continue to emphasize safety for most patients while also acknowledging that ICU cases may need deviations from package insert recommendations. Clinicians must be aware of the rationale behind each system’s approach to avoid misapplication. In conclusion, the piperacillin case illustrates ways divergent dosing philosophies can influence practice. The Japanese perspective emphasizes caution: high-dose regimens from the Sanford Guide should not be applied indiscriminately in general wards in Japan because they risk adverse effects and selection of resistant organisms. At the same time, clinicians should recognize that ICU contexts may legitimately require more aggressive dosing. Awareness of these nuances will enhance antimicrobial stewardship and promote safer, more rational antibiotic use across health care systems.

## Conclusions

The discrepancies in piperacillin dosing between the Sanford Guide and the Japanese package insert reflect fundamentally different philosophies of antimicrobial use. Japan has historically prioritized patient safety and minimization of adverse effects, whereas international guidelines―particularly those targeting patients who are critically ill―emphasize achieving optimal PK/PD targets even at the expense of higher drug exposure. Our analysis indicates that adopting Sanford-level regimens in Japanese general wards carries a substantial risk of drug-related toxicity and selection of resistant organisms. Conversely, adherence to the Japanese package insert in ICU settings may be insufficient for treating severe *Pseudomonas aeruginosa* infections. Therefore, clinicians in Japan should apply these dosing philosophies contextually: reserving aggressive regimens for ICU or high-risk cases while maintaining safety-focused dosing in general wards. Clearer communication of these principles will promote rational antimicrobial use, reduce ecological harm, and strengthen antimicrobial stewardship in Japan.

## Article Information

### Author Contributions

Osamu Uemura solely contributed to the conception, design, interpretation, and writing of this manuscript.

### Conflicts of Interest

None
